# Rapid detection of *Mycoplasma pneumoniae* CARDS toxin in clinical respiratory specimens by a loop-mediated isothermal amplification assay

**DOI:** 10.3389/fcimb.2025.1496829

**Published:** 2025-03-05

**Authors:** Yun Fang, Panpan Xie, Xin Zhang, Yue Zhang, Ning Yang, Yinghui Shi, Ruixia Xin, Yunbiao Bai, Wenkai Niu, Xin Yuan

**Affiliations:** ^1^ Department of Respiratory and Critical Care Medicine, Senior Department of Infectious Diseases, the Fifth Medical Center of PLA General Hospital, Beijing, China; ^2^ Senior Department of Infectious Diseases, The Fifth Medical Center of PLA General Hospital, National Clinical Research Center for Infectious Diseases, Beijing, China; ^3^ The Fifth Clinical Medical College, Anhui Medical University, Hefei, Anhui, China

**Keywords:** community-acquired respiratory distress syndrome toxin, detection methods, loop-mediated isothermal amplification assay, *Mycoplasma pneumoniae*, MP pneumonia

## Abstract

In light of the absence of rapid and precise diagnostic laboratory tests for the detection of *Mycoplasma pneumoniae* (MP), a prominent etiological agent implicated in a range of respiratory infections, we developed and evaluated a rapid and straightforward loop-mediated isothermal amplification (LAMP) assay targeting the MP community-acquired respiratory distress syndrome toxin (CARDS TX) gene. The LAMP assay was performed at 65°C for a duration of 60 min, yielding a minimum detection concentration of MP CARDS TX at 0.4986 pg/μl. The assay exhibited no cross-reactivity with 13 other prevalent pathogens associated with respiratory infections or with other common bacterial toxin genes. To further substantiate the validity of the LAMP assay, 200 pharyngeal swabs or bronchoalveolar lavage (BAL) samples were collected from inpatients diagnosed with community-acquired pneumonia (CAP) between June 2021 and July 2022. The results were compared with those obtained by the quantitative real-time polymerase chain reaction (qPCR) method for verification purposes. Of the 200 clinical specimens, 11 exhibited positive results for MP by LAMP and 10 displayed positive results for MP by qPCR (*P* = 1.000). In summary, a sensitive, specific, straightforward, and expeditious LAMP method for CARDS TX identification was developed to facilitate rapid detection of MP in point-of-care settings. This assay enables early and accurate diagnosis, even in resource-limited environments, which is important for proper antibiotic treatment and prognosis of MP infection.

## 1 Introduction

*Mycoplasma pneumoniae* (MP) is one of the most prevalent pathogens responsible for respiratory diseases. It is estimated to account for 10%–30% of cases of community-acquired pneumonia (CAP) (Atkinson et al., 2008). In addition to causing acute respiratory infections, MP can also induce chronic inflammatory responses in the respiratory tract, thereby contributing to the pathogenesis of chronic respiratory disorders such as asthma (Liu et al., 2021). Moreover, MP infections have been observed to result in damage to extrapulmonary organs, with encephalitis and myocarditis among such manifestations (D’Alonzo et al., 2018; Wandro et al., 2018; Poddighe, 2020; Aizawa et al., 2021; Katsumi et al., 2021; Wang et al., 2021). Although *M. pneumoniae* infection is typically self-limiting, severe *M. pneumoniae* pneumonia has been increasingly reported in recent years (Cillóniz et al., 2016; Waites et al., 2017; Ha et al., 2023; Lai et al., 2024). Despite extensive research, the precise pathogenic mechanism of MP remains unclear (Jiang et al., 2021). Due to the absence of a cell wall, MP is inherently resistant to β-lactam antibiotics (Waites et al., 2017; Lee et al., 2018). Furthermore, MP is responsible for regional outbreaks of epidemics with a periodicity of 2–6 years and presents with atypical signs and symptoms (Sauteur et al., 2024). Consequently, an early and accurate diagnosis is of great importance for the antibiotic treatment and prognosis of MP infection. *In-vitro* MP culture is a challenging and time-consuming process, serological methods require confirmation approximately one to two weeks after infection, and polymerase chain reaction (PCR) methods necessitate the utilization of expensive thermal cycling apparatus and well-established laboratories, which are challenging to implement in primary hospitals in regions with limited healthcare resources and infrastructure (Loens and Ieven, 2016; Waites et al., 2017). It is therefore imperative that a flexible and rapid method for field-deployable diagnostic tests for MP infections be developed.

The community-acquired respiratory distress syndrome toxin (CARDS TX), initially identified and named by Kannan et al, 2005, is a distinctive human surfactant protein A binding protein of MP. It plays a pivotal role in the pathogenesis of acute and chronic diseases associated with MP infection (Kannan et al., 2005; Maselli et al., 2018). In addition to promoting adhesion between MP and host target cells, CARDS TX also enters and diffuses into host cells by binding to cell surface receptors, thereby triggering the release of inflammatory factors that produce cytopathic effects. These effects ultimately result in a series of pathological changes (Su et al., 2021). Monitoring CARDS TX is of significant clinical importance for diagnosing MP infections and reflects the condition of MP pneumonia.

Loop-mediated isothermal amplification (LAMP) technology is a relatively new isothermal amplification technique for specific nucleic acid fragments initially described by Japanese researcher Notomi in 2000 (Mori and Notomi, 2020). The procedure is rapid and efficient, and the reaction can be accomplished in approximately 30–60 min. The complete amplification process necessitates only a thermostat and a timer. LAMP technology has gained considerable attention in recent years due to its advantageous characteristics, including rapidity, sensitivity, simplicity, good specificity, and low cost. The method has been successfully applied in the detection of pathogenic microorganisms, the identification of single-nucleotide polymorphisms, the surveillance of infectious disease, and other fields of scientific investigation (Cai et al., 2019). While there are existing methods for the detection of MP based on the LAMP method, there is currently no simple and rapid method targeting CARDS TX for clinical use. The development of a rapid test for the CARDS TX of MP based on the LAMP method has the potential to both clarify the diagnosis of MP infection and assist in the determination of the condition and prognosis.

This study aims to develop a rapid detection method for the CARDS TX based on the LAMP method, thus enabling more expedient diagnosis and assessment of MP infection.

## 2 Materials and methods

### 2.1 Preparing the target DNA

The QIAamp DNA Mini Kit (QIAGEN, Hilden, Germany) was utilized to extract genomic DNA from MP strains or samples, according to the manufacturer’s instructions. The CARDS TX plasmid was synthesized by Beijing Liuhe Huada Gene Technology Co., Ltd. (Beijing, China). The DNA concentrations were determined at OD260 by use of an ND-1000 spectrophotometer (Nano Drop Technologies, Inc., Wilmington, DE, USA).

### 2.2 Design of primers and probes

The MP standard reference strain sequence (ATCC15531) was obtained from the NCBI database. Four sets of LAMP primers for the CARDS TX gene were designed using Primer Explorer V5. Each primer set comprises four essential primers: forward internal primer (FIP), reverse internal primer (BIP), external forward primer (F3), and external reverse primer (B3), and/or two loop primers: forward loop primer (LF) and backward loop primer (LB). Additionally, three sets of quantitative real-time PCR (qPCR) primers were designed for the CARDS TX gene. All the LAMP and qPCR primers were synthesized by Beijing Liuhe Huada Gene Technology Co., Ltd (**Table 1** and **Supplementary Table S1**).

**Table 1:** LAMP and qPCR primer sequences designed and used.

Primers	Sequences (5’–3’)	Length (bp)
Primer set CARDS TX-28 used for LAMP		
CARDS TX-FIP	CGCATTAAATCTAACAAGTTCTCCCGGGCTTACTTATATGAAATTCGTG	49
CARDS TX-BIP	GACAAGTAGTATTTGACTCTGGTGAGGAAGTGCGTAAAGCTCTA	44
CARDS TX-LF	GCATTGTAAAAGTGTTGGTCGG	22
CARDS TX-LB	TCGAGAAATGGCACAAATGGG	21
CARDS TX-F3	TGGTTACGGGAATATGTACC	20
CARDS TX-B3	CCATCGGTAAACCATTCAC	19
Primer set CARDS TX-S used for qPCR		
CARDS TX-F	TGCTGTTCCCGTTGAACCTG	20
CARDS TX-R	CATTGGCTTGGGTTTGCAGC	20
CARDS TX-P	FAM-TCACCACCCGGCTGGTCGTGTTGTAGA- TAMRA	27

Sequences of primer sets specific to the CARDS TX gene of *M. pneumoniae* used for LAMP and qPCR in this study. CARDS TX-FIP, forward inner primer for LAMP; CARDS TX-BIP, backward inner primer for LAMP; CARDS TX-LF, loop forward for LAMP; CARDS TX-LB, loop backward for LAMP; CARDS TX-F3, outer forward primer for LAMP; CARDS TX-B3, outer backward primer for LAMP; CARDS TX-F, forward primer for qPCR; CARDS TX-R, reverse primer for qPCR; CARDS TX-P, probe primer for qPCR.

### 2.3 Development of CARDS TX LAMP

The DNA sample of the MP standard reference strain (ATCC15531) was extracted and employed as a positive control to establish the standard LAMP assay. The total volume of the LAMP assay reaction was 25 μl, the conditions of the reaction were 65°C for 60 min, and the reaction system was 12.5 μl of the reaction mixture (20 mmol/L Tris-HCl (pH 8.8) (Easobio, Beijing, China), 10 mmol/L KCl (HUSHI, Shanghai, China), 0.1% Tween 20 (Amresco, WA, USA), 10 mmol/L (NH4)2SO4 (HUSHI), 0.8 mol/L betaine (Sigma, St. Louis, MO, USA), 8 mmol/L MgSO4 [GUANGFU, Tianjin, China), and 1.4 mmol/L dNTPs (TIANGEN, Beijing, China)], 2.6 μl of the primer mixture (40 pmol for FIP and BIP, 5 pmol for F3 and B3, and 20 pmol for LF and LB), 0.5 μl of EveGreen (20× in water; Yeasen, Shanghai, China), 1 μl of Bst DNA polymerase (8000 units; New England BioLabs, Ipswich, MA, USA), 7.4 μl of deionized water, and 1 μl template DNA.

### 2.4 Specificity and sensitivity test of LAMP in the detection of CARDS TX

To investigate the specificity of the LAMP primers designed for CARDS TX, 13 common clinical respiratory pathogens and 3 bacterial toxins were detected by the LAMP assay. Clinical isolates of *Klebsiella pneumoniae*, *Serratia marcescens*, *Klebsiella oxytoca*, *Pseudomonas aeruginosa*, *Enterobacter aerogenes*, *Staphylococcus aureus*, *Enterobacter cloacae*, *Acinetobacter baumannii*, *Escherichia coli*, and *Proteus mirabilis*, confirmed by culture and sequencing, were selected. *Streptococcus pneumoniae* (BN 338425), *Branhamella catarrhalis* (BN 3375500), and *Haemophilus influenzae* (BN 337544) were purchased from Shenzhen Yibaishun Technology Co., Ltd. (Shenzhen, China). Plasmids containing Escherichia coli Shiga toxin, Staphylococcus aureus enterotoxin, and pertussis toxin were synthesized by Beijing Liuhe Huada Gene Technology Co., Ltd. The reference sequence for the three toxin genes was shown in **Supplementary Table S2**. The concentrations of DNA utilized for specific detection were all within the range of 40–60 pg/μl. Each test was performed in triplicate for each strain, toxin, and CARDS TX. To ascertain the sensitivity of the detection system, the CARDS TX plasmid was diluted 10 folds to obtain the CARDS TX plasmid with a concentration range of 49.86 ng/μl to 0.004986 fg/μl. The assay was repeated three times for each concentration, and the minimum consistent detection concentration of the three results was considered the minimum detection concentration for this method.

### 2.5 Clinical sample collection

A total of 200 patients with suspected CAP were selected from those with acute respiratory infections hospitalized in the Department of Respiratory and Critical Care Medicine, Senior Department of Infectious Diseases, the Fifth Medical Center of PLA General Hospital between June 2021 and July 2022. Patients with suspected CAP were identified based on the presence of certain signs (including a temperature above 38.0°C or below 36.0°C, as well as the presence of rales on lung auscultation) and symptoms (a new or worsening cough or dyspnea) (Wunderink and Waterer, 2017). A total of 122 throat swabs and 78 bronchoalveolar lavage fluid (BALF) specimens were collected. These samples were preserved in mycoplasma transport solution (*Mycoplasma* liquid medium CM0403B, additive G SROO59C, glucose, and sterile distilled water) and frozen at −80°C. All samples were collected with the informed consent of the patients. This research was reviewed and approved by the Ethics Review Group of the Fifth Medical Center of the PLA General Hospital (KY-2022-3-13-1).

### 2.6 Clinical samples were tested using the LAMP and qPCR methods

Clinical samples of BALF and throat swabs were tested using the LAMP method described in Section 2.3 with qPCR method as controls. Primers and probes for qPCR for CARDS TX were designed, and the optimal primers were screened. The instrument used was Bio-Rad CFX96 (Bio-Rad, CA, USA). The reaction system was 1 μl of template DNA, 1 μl of forward primer, 0.5 μl of TaqMan probe primer, 1 μl of reverse primer, 12.5 μl of Premix Ex Taq (Probe qPCR) (2 × Conc., TaKaRa, Kyoto, Japan), and 9 μl of deionized water. Conditions of the reaction were 95°C for 30 s, then 95°C for 30 s, and 62.8°C for 30 s for 40 cycles.

### 2.7 Statistical analysis

The two methods of LAMP assay and qPCR were compared by chi-square test and symmetric measure using SPSS software version 22 (SPSS Inc., San Francisco, CA, USA), and *P* < 0.05 was considered statistically significant.

## 3 Results

### 3.1 Screening the best LAMP primers

**Figure 1:**
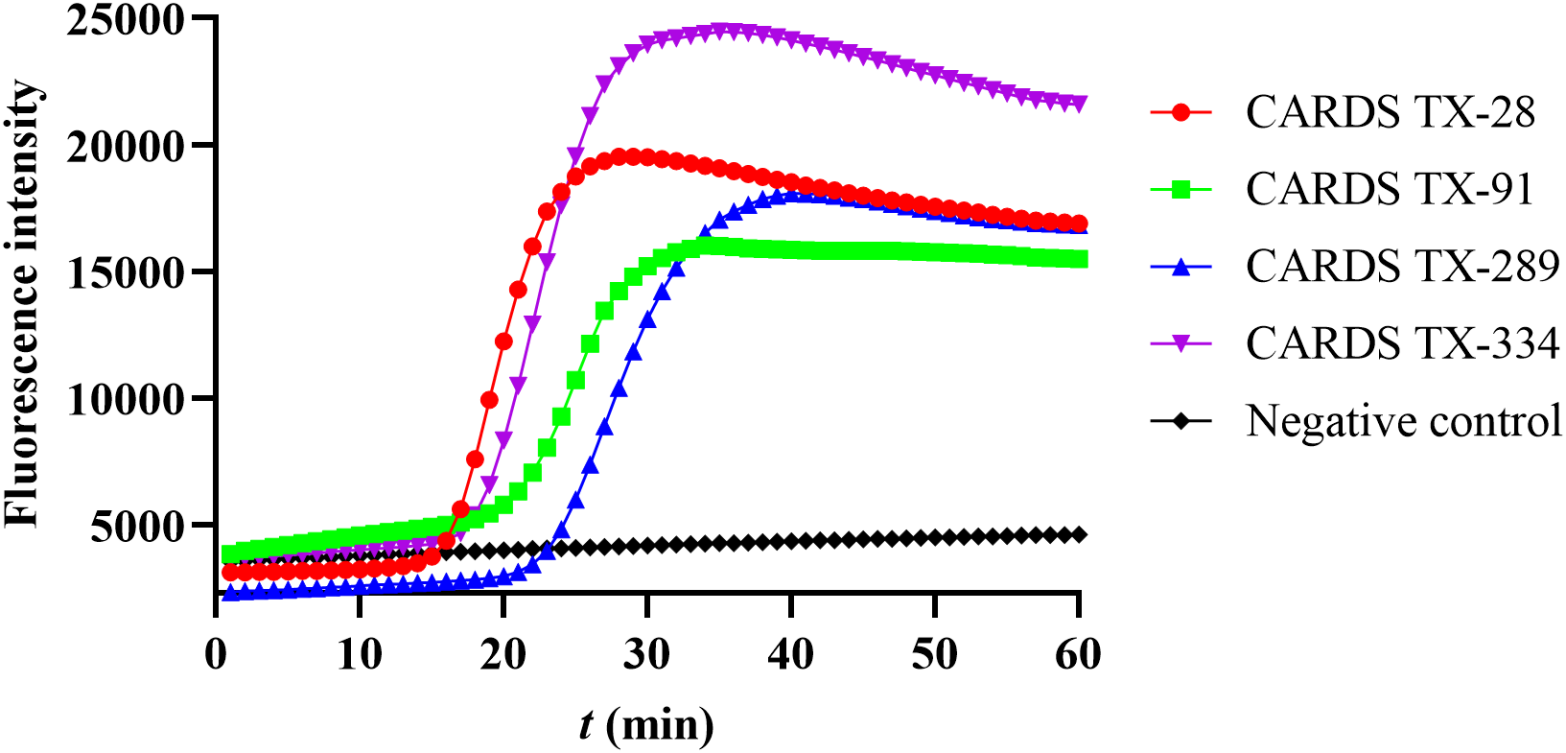
Screening the best LAMP primers for the detection of community-acquired respiratory distress syndrome toxin (CARDS TX). The number CARDS TX-28, 91, 289, and 334 represents four sets of LAMP primers designed to detect CARDS TX.

Four sets of LAMP primers have been designed and tested for the detection of CARDS TX. The LAMP reaction system containing the primer set CARDS TX-28 started amplification at 15.72 minutes and reached its peak at 28 min. In contrast, the reaction system containing primer sets CARDS TX-334, CARDS TX-91, and CARDS TX-289 initiated the amplification at 18, 21.89, and 23.7 min, respectively, and peaked at 35, 34, and 40 min. The primer set CARDS TX-28, which exhibited the earliest LAMP reaction, was deemed the most optimal (**Figure 1**).

### 3.2 Specificity and sensitivity results of LAMP detection of CARDS TX

**Figure 2:**
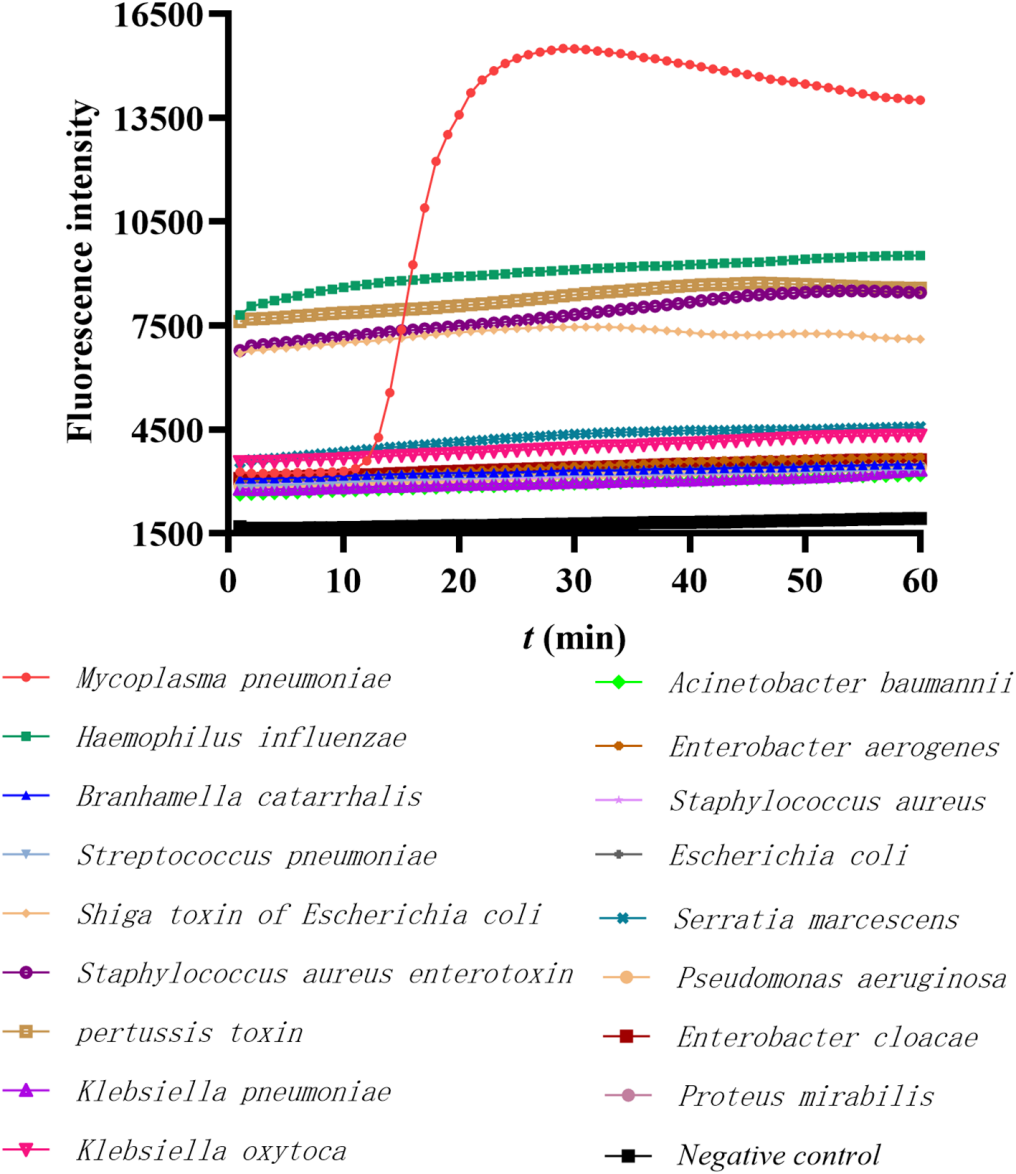
The specificity of the LAMP method in detecting MP FH. The red line represents the MP positive control, the black line represents the negative control, and others represent the DNA templates of 16 common respiratory pathogens or toxins.

To test the specificity of this primer set CARDS TX-28, 16 DNA or toxin genes from pathogenic microorganisms which commonly cause respiratory infections were used. The primer set demonstrated the inability to amplify other target genes, with the exception of the CARDS TX of MP, indicating that the detection method exhibits great specificity (**Figure 2**).

**Figure 3:**
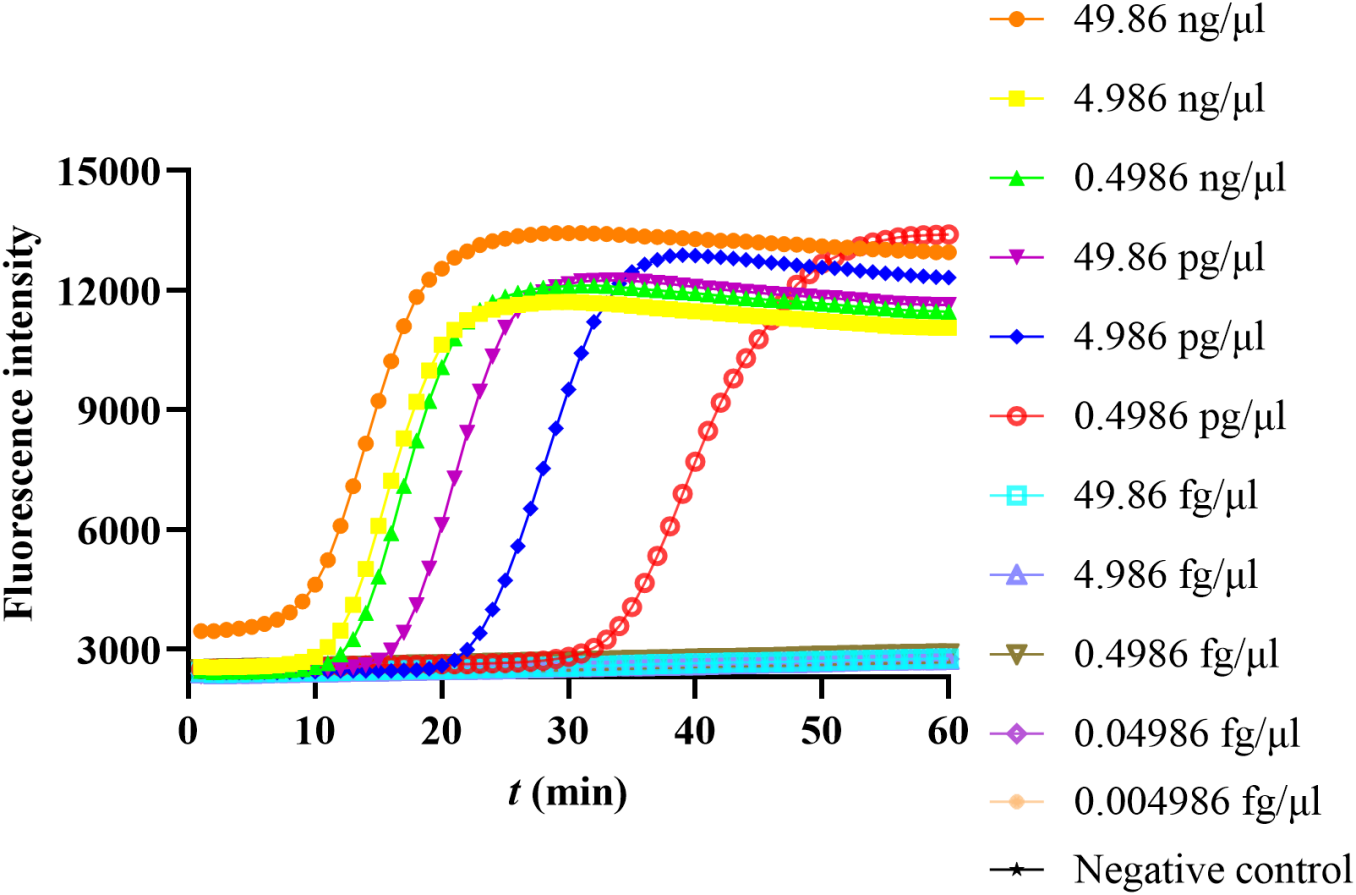
The sensitivity of the LAMP method in detecting community-acquired respiratory distress syndrome toxin (CARDS TX). The concentrations on the right indicate the continuous dilution concentrations of the CARDS TX plasmid.

To determine the sensitivity of this method, the continuously diluted CARDS TX plasmid was used as a template for repeated determination 3 times, and the minimum detection concentration was 0.4986 pg/μl (**Figure 3**).

Furthermore, the specificity and sensitivity of the remaining three primer sets (CARDS TX-91, CARDS TX-289, and CARDS TX-334) were determined using the aforementioned method. The results of the specificity test demonstrated that all three primer sets were capable of amplifying the CARDS TX of MP exclusively (**Supplementary Figure S1**). However, the minimum detection concentration of these three primer sets that could be detected was 5.28 pg/μl (**Supplementary Figure S2**), indicating that their sensitivity was considerably lower than that of the primer set CARDS TX-28.

### 3.3 Detection of clinical samples by the LAMP and qPCR methods

To ascertain the viability of clinical implementation, clinical specimens from 200 patients were collected, of which 122 were pharyngeal swab specimens and 78 were BALF specimens. All specimens were subjected to testing for CARDS TX using the LAMP and qPCR methods, with three replicates. The qPCR method, which has high sensitivity and specificity, was employed as the gold standard. The LAMP assay yielded 11 positive results (6 from BALF specimens and 5 from pharyngeal swabs) and 189 negative results, while the qPCR method produced 10 positive results (6 from BALF and 4 from pharyngeal swabs) and 190 negative results. The results showed that there was no statistically significant difference between the LAMP and qPCR methods for the detection of CARDS TX (*P* = 1.000). Symmetric measure demonstrated strong consistency of 95% between the two methods, as indicated by a Kappa value of 0.950 and a *P*-value of 0.001 (**Table 2**). Furthermore, all specimens were tested for nucleic acid in the clinical laboratory of our hospital, using the commercial qPCR kit targeting the MP 16Sr RNA (Sansure Biotech Co., Ltd, Changsha, China). The results were consistent with the results of the qPCR method targeting CARDS TX.

**Table 2:** Comparison of LAMP and qPCR methods for MP detection in clinical samples. .

Detection method	Clinical sample		*P*-value	Kappa value	*P*’ value
	Positive	Negative			
LAMP	11	189	1.000	0.950	0.001
qPCR	10	190			

*P*-value, chi-square test; *P*’ value, symmetric measure.

## 4 Discussion

This study describes the establishment of a LAMP method for the rapid, straightforward, sensitive, and specific detection of CARDS TX in MP. In comparison to the qPCR method, the detection performance of this method is not statistically significantly different, and the two methods demonstrated a high degree of concordance of 95%.

MP infection is a leading cause of a range of respiratory illnesses, including bronchitis, pneumonia, and asthma, as well as extrapulmonary conditions such as myocarditis and encephalitis (Fan et al., 2023; Zhu et al., 2024). It can cause regional epidemics or even pandemics, in which patients may exhibit no recognizable typical features or present with cases of severe disease (Sauteur et al., 2024; Cillóniz et al., 2016; Waites et al., 2017; Ha et al., 2023; Lai et al., 2024). Macrolides are a crucial class of antibiotics for the treatment of MP infections. However, in Asia, particularly in China and Japan, the macrolide resistance rate of MP exceeds 80% (Tanaka et al., 2017; Zhao et al., 2019; Wang et al., 2022a, Wang et al., 2022b). In these regions, fluoroquinolones and tetracycline antibiotics are more frequently recommended for the treatment of MP infections in adults. Furthermore, MP exhibits intrinsic resistance to β-lactams (Waites et al., 2017; Lee et al., 2018). The empirical use of β-lactams or macrolides in the absence of an identified etiology may result in ineffective treatment and a worsening of the patient’s condition. Timely diagnosis and precise treatment are therefore of paramount importance in the management of MP infection (Wang et al., 2023). Three principal methods are employed for the detection of MP: culture, serological, and PCR-based molecular biology. However, the culture and serological methods are time consuming, typically requiring at least 1 to 2 weeks, while the PCR method necessitates the availability of high-quality laboratory facilities (Loens and Ieven, 2016; Waites et al., 2017). Consequently, there is currently no straightforward and rapid method for bedside diagnosis of MP infection (Cai et al., 2019).

The LAMP technique has been employed for the detection of a range of pathogens due to its greater simplicity and lower cost in comparison to PCR. The technique is now recommended as the first-line diagnostic method for detecting acute MP infections in Japan (Saito et al., 2005; Kakuya et al., 2014). A LAMP assay targeting the P1 gene of MP has been established for the detection of MP in pharyngeal swabs (Wang et al., 2019), and there are also other clinical and rapid MP molecular assays from throat swabs-based targeting 16S rRNA or CARDS TX gene that are commercially available and FDA cleared (Waites et al., 2017). However, there are certain limitations to their use in a clinical setting. For instance, the performance of these methods has only been established in human pharyngeal swab specimens, or the conditions required for their use are more complex and challenging to implement in rapid, point-of-care testing. In some cases, it is challenging to determine whether a specimen from the upper respiratory tract is indicative of infection, colonization, or contamination (Peng et al., 2020; Gunasekaran et al., 2019). Bronchoalveolar lavage fluid (BALF) is a reliable specimen for the identification of pathogens associated with lower respiratory tract infections (Wang et al., 2020). In addition, it is noteworthy that Ieven et al. found that assays targeting the P1 gene were more sensitive than those targeting 16S rRNA (Ieven et al., 1996). Importantly, Peters et al. reported that the CARDS TX PCR was capable of detecting 10 times more patients with MP than a PCR utilizing the P1 protein gene as a target gene (Peters et al., 2011).

It has been demonstrated that CARDS TX plays a significant role in the pathogenesis of MP infection (Maselli et al., 2018; Li et al., 2019; Ramasamy et al., 2021). In the acute phase of MP infection, CARDS TX can aggravate the damage to the respiratory system by directly destroying respiratory epithelial cells or inducing an inflammatory response; in the chronic phase, the persistence of CARDS TX in the body can result in chronic airway inflammation, which may lead to the induction of asthma, acute exacerbations of chronic obstructive pulmonary disease (AECOPD), and other associated complications (Peters et al., 2011; Medina et al., 2012). Accordingly, the risk of developing asthma subsequent to MP infection can be evaluated by monitoring respiratory CARDS TX (Xu et al., 2024). In addition, there was a positive correlation between CARDS TX expression levels and clinical severity. A study demonstrated that the severity of MP infection was positively correlated with patients’ serum CARDS TX levels (Ma et al., 2018). Li et al. reported that refractory MP pneumonia had significantly higher levels of CARDS TX in alveolar lavage fluid than patients with nonrefractory MP (Li et al., 2019). It has also been found that CARDS TX persists in the body for more than 600 days and is associated with poor control of prolonged asthma (Peters et al., 2011). All these studies suggest that the detection of CARDS TX gene or protein is of considerable value in the diagnosis and evaluation of MP infection, especially in the early stage of infection, and not only this, but the concentration of CARDS TX may also indicate the clinical changes of MP pneumonia.

It is of significant clinical importance that the detection of CARDS TX in MP through the LAMP method serves not only for the diagnosis of MP infection but also for the estimation of the severity and prognosis of MP infection. The LAMP method utilized in this study to detect MP CARDS TX takes only one incubator, which is easy to operate and has the superiority of saving time (within 60 min). It is optimally suited for expeditious point-of-care testing and settings where medical resources are relatively scarce. Moreover, compared with the gold standard PCR method, there was no statistical difference in the detection efficiency. The LAMP reaction employed in this study utilized six oligonucleotide primers situated in disparate regions of the CARDS TX gene to amplify the target DNA. It is therefore highly specific. Furthermore, *in-silico* analysis of amino acid sequences indicated that CARDS TX shares high sequence similarity with the pertussis toxin S1 subunit from *Bordetella pertussis*. Compared to Xiao et al (Xiao et al., 2022), our method has the advantage of being tested for cross-reactivity against three additional toxins, including pertussis toxin, which validates the specificity of the method and makes the results more reliable. In a previous study, the PCR method targeting the MP CARDS TX gene demonstrated superior sensitivity compared to the PCR method targeting the P1 gene (Peters et al., 2011). This finding was further corroborated in the present study, in which the LAMP method was employed for the detection of CARDS TX, exhibiting a minimum detectable concentration of 0.4986 pg/μl. This value was markedly lower than that observed in the LAMP reaction targeting the P1 gene, as previously described by Wang et al (Wang et al., 2019). The aforementioned evidence substantiates the assertion that the methodology based on the LAMP reaction targeting MP CARDS TX, as delineated in this study, exhibits high sensitivity and specificity for the detection of MP infection. A further advantage of our work over that of Xiao et al (Xiao et al., 2022). is that the CARDS TX primers were designed for use with both LAMP and qPCR methods. The qPCR method targeting CARDS TX was used as the gold standard. This allows for a more direct comparison of the detection efficacy of the LAMP and PCR methods. The results of the two methods exhibited a high degree of consistency, with the qPCR method demonstrating a lower positive detection rate than the LAMP method. Furthermore, our established LAMP method has been successfully employed for the detection of BALF specimens, thereby further illustrating its broad clinical applicability. Therefore, this method is an effective detection method that has the potential to significantly impact clinical practice, particularly in settings with limited medical funding resources, enabling the monitoring of potential MP outbreaks and guiding diagnosis and treatment decisions.

This study is flawed in the following ways. First, the species of bacteria designed for specific detection was limited. Second, the number of clinical samples employed for comparison between the LAMP and qPCR methods was relatively small. Nevertheless, based on the available epidemiologic data, the majority of common bacteria and toxins susceptible to comorbid MP infections have been incorporated into the specific test, thereby rendering the use of this LAMP assay a feasible option for the diagnosis of respiratory tract infections in a broad range of clinical settings. Furthermore, the current method does not permit the quantification of CARDS TX in the specimens under examination. The monitoring of CARDS TX levels is a clinically significant objective, which can be achieved through the utilization of methodologies such as CRISPR/Cas12b-assisted LAMP and the quantitative LAMP colorimetric phenol red method. This is the direction we are going to delve into next.

## 5 Conclusion

Based on the LAMP technology and the CARDS TX gene, we have successfully established a method for rapid and accurate detection of MP. This method is characterized by its high specificity and sensitivity, as well as its simplicity and the ability to produce results that are discernible to the naked eye within less than an hour. The detection of CARDS TX in MP also facilitates the assessment of the severity and prognosis of MP infection. This assay enables early and precise diagnosis in various medical settings, especially for point-of-care testing and in resource-limited environments, which is crucial for proper antibiotic treatment and the prognosis of MP infection. The LAMP assay targeting MP CARDS TX is a promising tool for the early surveillance and diagnosis of MP infection.

## Data Availability

The raw data supporting the conclusions of this article will be made available by the authors, without undue reservation.
