# Supplementary material for: Rapid detection of Mycoplasma pneumoniae CARDS toxin in clinical respiratory specimens by a loop-mediated isothermal amplification assay
Source: Front Cell Infect Microbiol. 2025 Mar 5;15:1496829. doi: 10.3389/fcimb.2025.1496829 (PMC11962300; doi:10.3389/fcimb.2025.1496829)
Supplement: Supplementary file 1 [file DataSheet1.docx]

**Supplementary tables**

**Table S1.** LAMP and qPCR primer sequences designed and used

| **Primers** | **Sequences (5'-3')** | **Length (bp)** |
| --- | --- | --- |
| Primer set CARDS TX-91 used for LAMP | | |
| CARDS TX-FIP | ATCTACTAGTCAAGCACTACGGACACAACGTGAATGGTTTACCGA | 45 |
| CARDS TX-BIP | CGGCTGGTCGTGTTGTAGAGGCTCTTGATAATGAGGGTTG | 40 |
| CARDS TX-LF | GCTGCTGCAATTGGACCA | 18 |
| CARDS TX-F3 | CGCACTTCCTTTGCGTAT | 18 |
| CARDS TX-B3 | TCATTGGCTTGGGTTTGC | 18 |
| Primer set CARDS TX-289 used for LAMP | | |
| CARDS TX-FIP | CTTGGTATTTCAGAACAAGCCATTTGCAATAACTCAGTTTCGGGT | 45 |
| CARDS TX-BIP | GTCCTTTGAAAAATCCACCTAGTGACAAACTGCGCATGGGTAG | 43 |
| CARDS TX-LF | GTTTGACCTAACAGTTCAGAGGAG | 24 |
| CARDS TX-F3 | AATGACACCGCAAGACAG | 18 |
| CARDS TX-B3 | CATCAACAAAGAAGGTGCTAG | 21 |
| Primer set CARDS TX-334 used for LAMP | | |
| CARDS TX-FIP | GCTGAACATCAACAAAGAAGGTGCATTGTTGATGAATGTACTACCCA | 47 |
| CARDS TX-BIP | ATACCCCACAATTAAGTGGTTGATTCATAGAATATCTGTCCATCTGG | 47 |
| CARDS TX-LF | TGCACGCATAGTAACAAACTGC | 22 |
| CARDS TX-F3 | CCACCTAGTGATTTGGAAGA | 20 |
| CARDS TX-B3 | GGACAAAGAAGATTTTCGAAGTT | 23 |
| Primer set CARDS TX-2 used for qPCR | | |
| CARDS TX-F | TCGCGTGATGACCTTACCAT | 20 |
| CARDS TX-R | GTCAGCGTAACAGCGAATGT | 20 |
| CARDS TX-P | FAM-CCGTTGAAGGGCTTAACTTCCGCC- TAMRA | 24 |
| Primer set CARDS TX-8 used for qPCR | | |
| CARDS TX-F | CAGCTTCCGTTGCTGATGTT | 20 |
| CARDS TX-R | CTTCAATCAGGGCACGCAAA | 20 |
| CARDS TX-P | FAM-TCGGAAGGTACTTCCGCTTCGCT- TAMRA | 23 |

Sequences of primer sets specific to the CARDS TX gene of *M. pneumoniae* used for LAMP assay and qPCR assay in this study. CARDS TX-FIP, forward inner primer for LAMP; CARDS TX-BIP, backward inner primer for LAMP; CARDS TX-LF, loop forward for LAMP; CARDS TX-F3, outer forward primer for LAMP; CARDS TX-B3, outer backward primer for LAMP; CARDS TX-F, forward primer for qPCR; CARDS TX-R, reverse primer for qPCR; CARDS TX-P, probe primer for qPCR.

**Table S2.** The reference sequence for Shiga toxin, Staphylococcus aureus enterotoxin, and pertussis toxin genes.

| Shiga toxin sequence |
| --- |
| >AB048226.1 Escherichia coli genes for Shiga toxin 2 A-subunit, Shiga toxin 2 B-subunit, complete cds, strain: #S-7  ATGAAGTGTATATTGTTAAAATGGATACTGTGCCTGTTGCTGGGCTTTTCTTCGGTATCCTATTCCCGGGAATTTACGATAGACTTTTCGACTCAACAAAGTTATGTCTCTTCGTTAAATAGTATACGGACAGAAATATCGACCCCACTTGAACATATATCTCAGGGGACCACATCGGTGTCTGTTATTAACCACACCCCACCGGGCAGTTATTTTGCTGTGGATATACGAGGGCTTGATGTCTATCGGGCGCGTTTTGACCATCTTCGTCTGATTATTGAGCAAAATAATTTATATGTGGCCGGATTCGTTAATACGGCAACAAATATTTTCTACAGATTTTCAGATTTTGCACATATATCAGTGCCCGGTGTGACAACTGTTTCCATGACAACGGACAGCAGTTATACCACTCTGCAACGTGTCGCAGCGCTGGAACGTTCCGGAATGCAAATCAGTCGTCACTCACTGGTTTCATCATATCTGGCGTTAATGGAGTTTAGTGGAAATGCCATGACCAGAGATGCATCCAGAGCAGTTCTGCGTTTTGTCACTGTCACAGCAGAAGCCTTACGGTTCAGGCAAATACAGAGAGAATTTCGTCTGGCACTGTCTGAAACTGCTCCTGTTTATACGATGACACCGGAAGAAGTGGACCTCACACTGAACTGGGGGAGAATCAGCAATGTGCTTCCGGAGTTTCGGGGAGAGGGTGGTGTCAGAGTGGGGCGAATATCCTTTAATAATATATCAGCGATACTGGGCACAGTGGCGGTTATACTGAATTGCCATCATCAGGGGGCGCGTTCCGTTCGCGCCGTGAATGAAGAGATACAACCAGAATGTCAGATAACTGGCGACAGGCCAGTTATAAGGATAAACAATACTTTATGGGAAAGTAATACCGCAGCTGCTTTTCTGAATCGCAGGGCTCACTCTTTAAATACATCCGGAGAATAACAGGAGTTAAATATGAAGAAGATATTTGTAGCGGCTTTATTTGCTTTTGTTTCTGTTAATGCAATGGCAGCTGATTGTGCAAAAGGTAAAATTGAGTTCTCTAAGTATAATGAGAATGATACATTCACAGTAAAAGTGGCCGGGAAAGAGTACTGGACTAACCGCTGGAATCTGCAACCGCTACTGCAAAGCGCACAGTTAACAGGAATGACGGTAACAATCAAATCAAATACCTGTGCGTCAGGTTCAGGATTTGCTGAAGTGCAGTTTAATAATGACTGA |
| Staphylococcus aureus enterotoxin sequence |
| >L13379.1 Staphylococcus aureus enterotoxin gene, 3' end  GAGAGCCAACCAGACCCTACGCCAGATGAGTTGCACAAAGCGAGTAAATTCACTGGTTTGATGGAAAATATGAAAGTTTTATATGATGATCGTTATGTATCAGCAACTAAAGTTAAGTCTGTAGATAAATTTTTGGCACATGATTTAATTTATAACATTAGTGATAAAAAACTGAAAAATTATGACAAAGTGAAAACAGAGTTATTAAATGAAGATTTAGCAAAGAAGTACAAAGATGAAGTAGTTGATGTGTATGGATCAAATTACTATGTAAACTGCTGTTTTTCATCCAAAGATAATGTAGGTAAAGTTACAGGTGGTAAAACTTGTATGTATGGAGGAATAACAAAACATGAAGGAAACCACTTTGATAATGGGAACTTACAAAATGTACTTATAAGAGTTTATGAAAATAAAAGAAACACAATTTCTTTTGAAGTGCAAACTGATAAGAAAAGTGTAACAGCTCAAGAACTAGACATAAAAGCTAGGAGTTTTTTAATTAATAAAAAAAATTTGTATGAGTTTAACAGTTCACCATATGAAACAGGATATATAAAATTTATTGAAAATAACGGCAATACTTTTTGGTATGATATGATGCCTGCACCAGGAGATAAGTTTGACCAATCTAAATATTTAATGATGTACAACGACAATAAAACGGTTGATTCTAAAAGTGTGAAGATAGAAGTCCACCTTACAACAAAGAATGGATAA |
| Pertussis toxin sequence |
| >AJ245366.1 Bordetella pertussis s1 gene for pertussis toxin subunit S1 (s1A allele)  ATGCGTTGCACTCGGGCAATTCGCCAAACCGCAAGAACAGGCTGGCTGACGTGGCTGGCGATTCTTGCCGTCACGGCGCCCGTGACTTCGCCGGCATGGGCCGACGATCCTCCCGCCACCGTATACCGCTATGACTCCCGCCCGCCGGAGGACGTTTTCCAGAACGGATTCACGGCGTGGGGAAACAACGACAATGTGCTCGACCATCTGACCGGACGTTCCTGCCAGGTCGGCAGCAGCAACAGCGCTTTCGTCTCCACCAGCAGCAGCCGGCGCTATACCGAGGTCTATCTCGAACATCGCATGCAGGAAGCGGTCGAGGCCGAACGCGCCGGCAGGGGCACCGGCCACTTCATCGGCTACATCTACGAAGTCCGCGCCGACAACAATTTCTACGGCGCCGCCAGCTCGTACTTCGAATACGTCGACACTTATGGCGACAATGCCGGCCGTATCCTCGCCGGCGCGCTGGCCACCTACCAGAGCGAATATCTGGCACACCGGCGCATTCCGCCCGAAAACATCCGCAGGGTAACGCGGGTCTATCACAACGGCATCACCGGCGAGACCACGACCACGGAGTATTCCAACGCTCGCTACGTCAGCCAGCAGACTCGCGCCAATCCCAACCCCTACACATCGCGAAGGTCCGTAGCGTCGATCGTCGGCACATTGGTGCGCATAGCGCCGGTGATAGGCGCTTGCATGGCGCGGCAGGCCGAAAGCTCCGAGGCCATGGCAGCCTGGTCCGAACGCGCCGGCGAGGCGATGGTTCTCGTGTACTACGAAAGCATCGCGTATTCGTTCTAG |

**Supplementary figures**


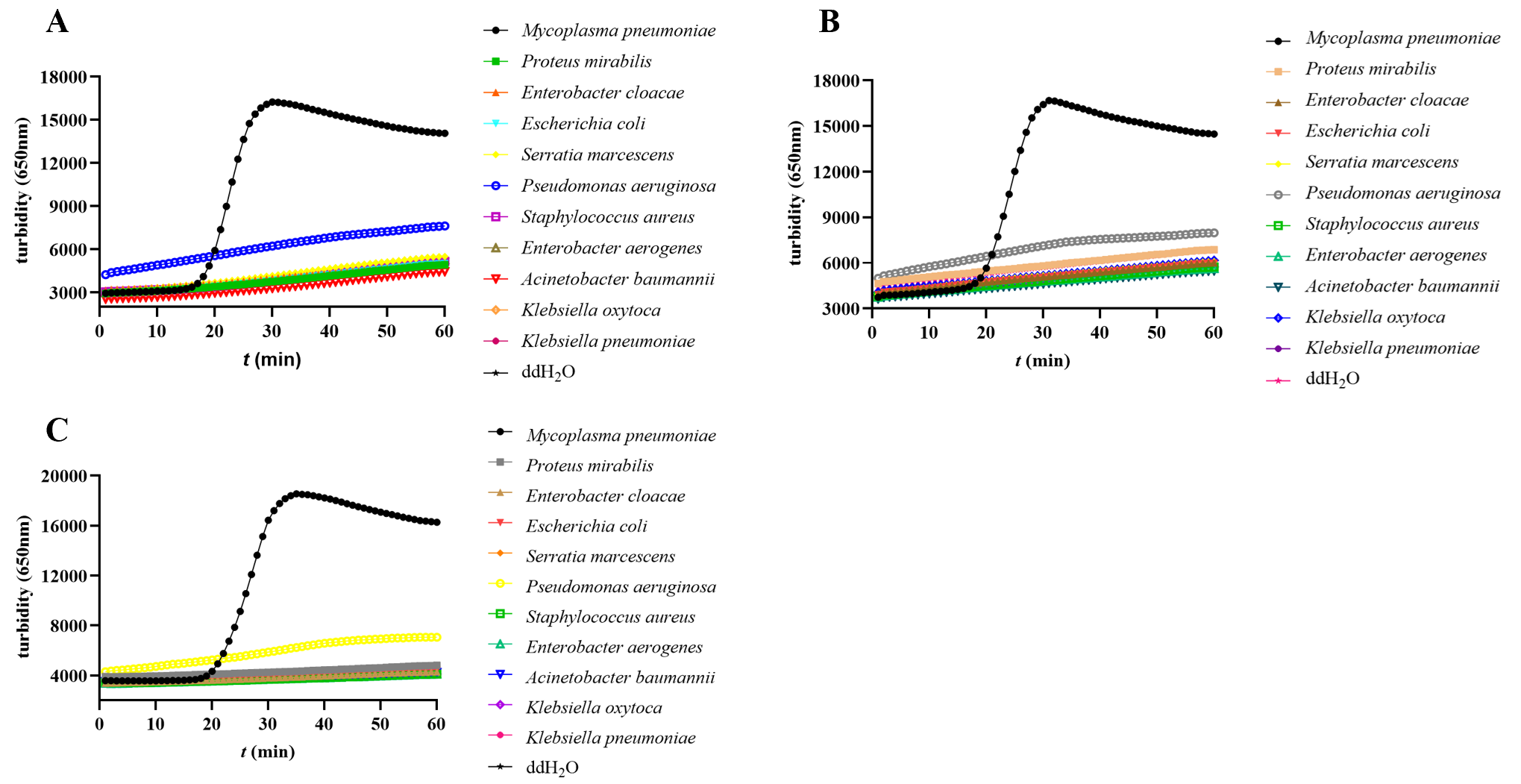


**Figure S1.** The specificity of the LAMP method in detecting MP FH. A, CARDS TX-91; B, CARDS TX-289; C, CARDS TX-334.


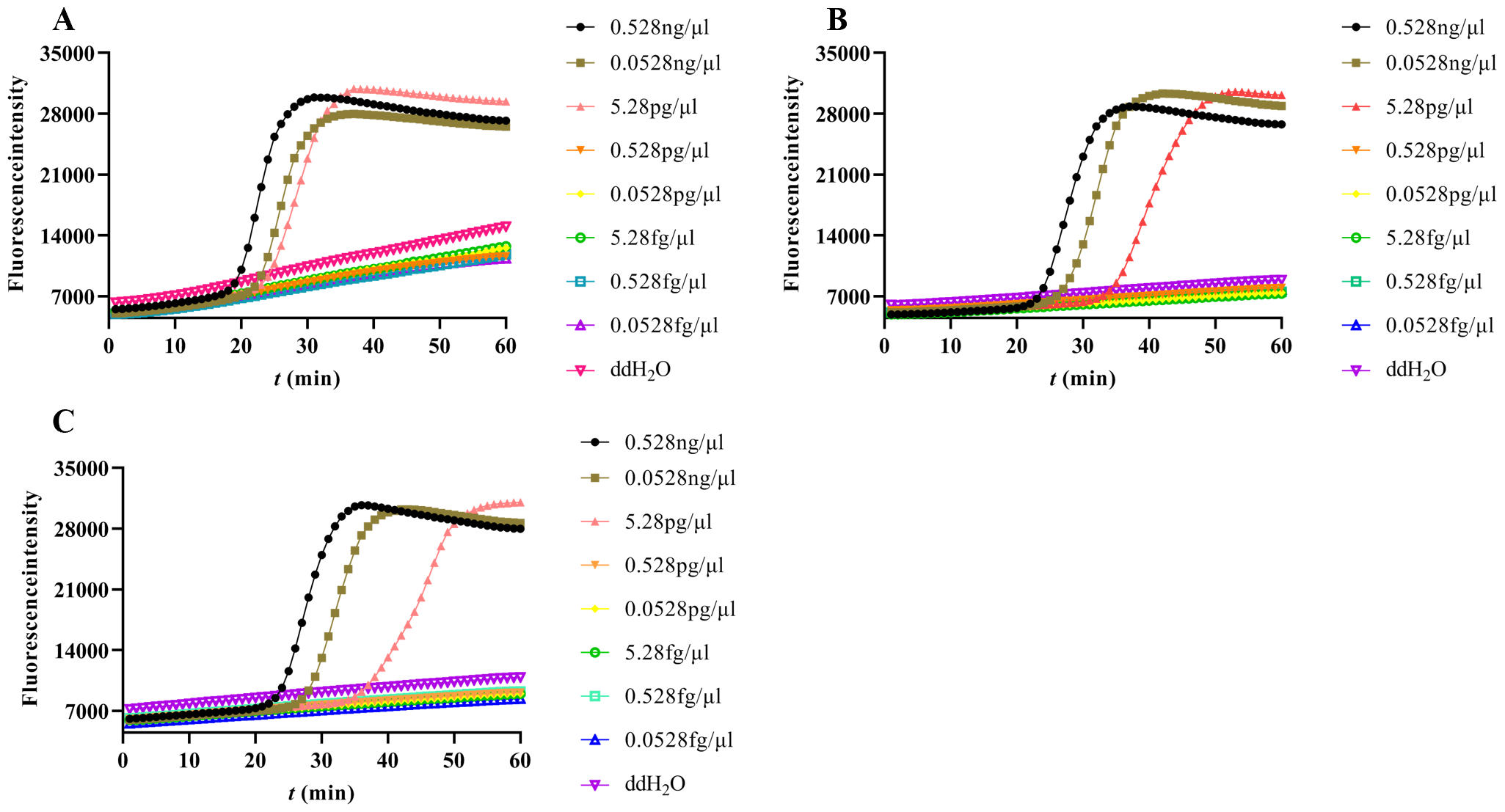


**Figure S2.** The sensitivity of the LAMP method in detecting CARDS TX. The concentrations on the right indicate the continuous dilution concentrations of the CARDS TX plasmid. A, CARDS TX-91; B, CARDS TX-289; C, CARDS TX-334.
